# Simultaneous Radial and Ipsilateral Ulnar Artery Compression versus Isolated Radial Artery Compression after Conventional Radial Access for Coronary Angiography and/or Intervention: A Systematic Review and Meta-Analysis

**DOI:** 10.3390/jcm11237013

**Published:** 2022-11-27

**Authors:** Francesco Condello, Michele Cacia, Matteo Sturla, Riccardo Terzi, Jorge Sánz-Sanchez, Bernhard Reimers, Gabriele L. Gasparini, Paolo Pagnotta, Sabato Sorrentino, Carmen Spaccarotella, Ciro Indolfi, Alberto Polimeni

**Affiliations:** 1 Department of Biomedical Sciences, Humanitas University, 20090 Milan, Italy; 2 IRCCS Humanitas Research Hospital, 20089 Milan, Italy; 3 IRCCS Ospedale Galeazzi-Sant’Ambrogio, University Cardiology Department, 20157 Milan, Italy; 4 Hospital Universitario y Politécnico La Fe, 46026 Valencia, Spain; 5 Centro de Investigacion Biomedica en Red Enfermedades Cardiovasculares (CIBERCV), 28029 Madrid, Spain; 6 Center for Cardiovascular Research, Magna Graecia University, 88100 Catanzaro, Italy; 7 Division of Cardiology, Department of Medical and Surgical Sciences, Magna Graecia University, 88100 Catanzaro, Italy; 8 Division of Cardiology, Department of Advanced Biomedical Science, Federico II University, 80138 Naples, Italy

**Keywords:** radial access, radial artery occlusion, hemostasis, coronary angiography, percutaneous coronary intervention

## Abstract

Background: Simultaneous ulnar and radial artery compression (SURC) has emerged as a strategy to increase radial artery flow and mitigate radial artery occlusion (RAO) while achieving adequate hemostasis after transradial access (TRA), though its technical adoption has been limited worldwide. Methods: A systematic search of studies comparing SURC versus isolated radial artery compression after TRA for coronary angiography and/or intervention was performed. Data were pooled by meta-analysis using random-effects models. Odds ratios (OR) with relative 95% confidence intervals (CI) and standardized mean difference were used as measures of effect estimates. The primary endpoint was the occurrence of overall RAO. Results: A total of 6 studies and 6793 patients were included. SURC method as compared to isolated radial artery compression was associated with a lower risk of RAO both overall (OR 0.29; 95% CI, 0.13–0.61, *p* < 0.001; number needed to treat to benefit [NNTB] =38) and in-hospital (OR 0.28; 95% CI: 0.10 to 0.75; *p* = 0.01, NNTB = 36), with a reduced risk of unsuccessful patent hemostasis (OR: 0.13; 95% CI: 0.02 to 0.85; *p* = 0.03, NNT = 5) and upper extremity pain (OR: 0.48; 95% CI: 0.24 to 0.95; *p* = 0.04, NNTB = 124). No significant difference was observed in hemostasis time and in the risk of hematoma. Conclusion: Compared to isolated radial artery compression, SURC is associated with lower risk of RAO, unsuccessful patent hemostasis, and reported upper limb pain, without any trade-off in safety outcomes. With further development of dedicated dual compression devices, the proposed technique should be freed from usage constraints.

## 1. Introduction

Radial artery occlusion (RAO) is the most common structural complication of transradial access (TRA), with a reported incidence of 7.7% 24 h after the procedure but reaching as high as 30% in some studies [1,2,3,4]. RAO itself has not been associated with clinically overt ischemic complications owing to the presence of numerous and extensive anastomotic vascular connections in the hand, and prior studies did not find a relationship between RAO and the occurrence of hand motor functional impairment or instrumental signs of ischemia [5,6,7]. Nevertheless, the occurrence of RAO limits future percutaneous cardiovascular procedures through the same access site, reduces the availability of conduits for coronary artery bypass grafting surgery and arteriovenous fistula preparation in patients requiring hemodialysis, and may limit future intra-arterial pressure monitoring [8]. Furthermore, as more complex procedures are being performed by TRA, increased use of large bore sheaths will further impact the incidence of RAO [9]. Several pharmacological and nonpharmacological strategies have been shown to lower the risk of RAO and are recommended as best practice by consensus documents. These include the use of the lowest profile system necessary to complete the procedure, adequate pre- and post-procedural anticoagulation and vasodilator administration, non-occlusive hemostasis with a minimal pressure strategy, and shorter hemostasis time. Finally, systematic assessment of radial artery patency before discharge has been shown to lower the risk of RAO, and the assessment of patency at follow-up visit should be performed [10,11].

Despite these best practice recommendations, the reported incidence of RAO right now remains much higher than the target that, according to the experts’ opinion, should be pursued in each center where a TRA program is implemented (RAO incidence < 5% at 24 h after the procedure) [1,3,10]. The augmentation of blood flow in the radial artery through simultaneous ipsilateral ulnar and radial artery compression (SURC) has emerged as a potential strategy to achieve patent hemostasis in a larger number of patients and to prevent RAO [12]. Increased radial artery blood flow by SURC allows the compressed radial artery to be forced open while promoting localized fibrinolysis.

This study aimed to provide a quantitative and comprehensive assessment of the effects of the SURC method versus isolated radial artery compression after conventional radial access for coronary angiography and/or percutaneous coronary intervention (PCI).

## 2. Materials and Methods

This meta-analysis was performed according to the updated Preferred Reporting Items for Systematic Reviews and Meta-Analyses (PRISMA) 2020 guidelines [13].

### 2.1. Data Source and Search Strategy

Two reviewers (FC, MS) independently identified the relevant studies by an electronic search of the PubMed/MEDLINE, Scopus, Cochrane Central Register of Controlled Trials, and ClinicalTrials.gov databases (from inception to August 2022). The following search terms and keywords were used individually or in combination with each other: “radial”, “occlusion”, “thrombosis”, “RAO”, “ulnar”, “ipsilateral”, “hemosta*”, “compress*”. No language, publication date, or publication status restrictions were imposed. The references of the included studies, published systematic reviews, meta-analyses and editorials were also screened. This systematic review was registered on PROSPERO with the following registration number: CRD42022354386.

### 2.2. Study Selection

Two reviewers (FC, MS) independently assessed trial eligibility on the basis of titles, abstracts, and full-text reports. Discrepancies in the study selection were discussed and resolved with another reviewer (AP). Eligible trials had to satisfy the following pre-specified criteria: (1) studies that compared SURC method versus isolated radial artery compression for patent hemostasis of the radial artery; (2) inclusion of patients undergoing coronary angiography and/or PCI via TRA. Exclusion criteria were (1) comparison of techniques other than SURC to prevent RAO; (2) lack of reported clinical outcome data; (3) overlapping patient populations.

### 2.3. Data Extraction and Quality Assessment

Two reviewers (FC, MS) independently extracted data (i.e., baseline characteristics, inclusion and exclusion criteria, the definition of outcomes, numbers of events) from eligible studies using a standardized data abstraction form and independently entered outcome data into a Microsoft Excel spreadsheet (2016 version). Another reviewer (AP) then manually cross-checked these, referring to the original source data when discrepancies were identified. Any disagreements regarding collected information between the two reviewers were reconciled through discussion with a third reviewer (AP). Two reviewers (FC, MS) independently and systematically assessed randomized controlled trials (RCT) methodological quality using the revised Cochrane risk-of-bias tool (RoB 2.0), assessing five domains of bias for each outcome: randomization process; deviation from intended interventions; missing outcome data; measurement of the outcome; and selection of the reported results [14]. Risk of bias summary reported each risk-of-bias item for each included study. Any disagreement was resolved with a third reviewer (AP). Risk-of-bias assessment for non-randomized studies was performed using the Risk Of Bias In Non-randomized Studies of Interventions (ROBINS-I) tool assessing seven domains of bias for each outcome: confounding, selection of participants into the study, classification of interventions, deviation from intended interventions, missing data, measurement of outcomes, selection of the reported results [15]. A risk of bias summary reporting each risk-for-bias item for each included study was reported. Any disagreement was resolved with a third reviewer (AP).

### 2.4. Outcome Measures

The primary endpoint of this study was the occurrence of forearm RAO. In the main analysis, RAO at the most extended follow-up available in each study was considered for inclusion and defined as overall RAO. Secondary endpoints were as follows: in-hospital forearm RAO, unsuccessful patent hemostasis, local hematoma defined as Early Discharge After Transradial Stenting of Coronary Arteries (EASY) or modified EASY hematoma ≥ I, hemostasis time and upper extremity pain. Endpoints were attributed according to the definition and timing used in each study.

### 2.5. Statistical Analysis

For dichotomous outcomes, odds ratios (ORs) with 95% confidence intervals (CIs) were calculated from the available data. Trial-specific ORs were combined using the DerSimonian and Laird random-effects model due to the presence of heterogeneity. [16] For continuous outcomes, the effect size was computed as the Hedges’s standardized mean difference (SMD), and trial-specific effect sizes were pooled with a random-effects model due to the presence of heterogeneity with the use of the Sidik–Jonkman method to estimate the between-study variance τ^2^ [17,18]. The number of patients needed to treat for an additional beneficial outcome (NNTB) and the number needed to treat for an additional harmful outcome (NNTH) were calculated from weighted estimates of pooled ORs from the random-effects meta-analytic model, using the macro “metannt,” as: 1/(projected control group event rate—projected treatment group event rate). The corresponding 95% CI was calculated using the 95% CI of the effect size applied to the control group event rate. We also calculated trial-specific absolute risk differences with 95% CI for each endpoint and reported the number of events avoided or caused per 1000 patients treated with the 95% CI. The presence of heterogeneity among studies was evaluated with the Cochran Q chi-square test with *p* ≤ 0.10 considered to indicate statistical significance, estimating the between-studies variance tau-square and using the I^2^ test to evaluate inconsistency [19]. The I^2^ statistic is derived from the Q statistic ([Q − df/Q] × 100) and describes the percentage of total variation across studies that is due to heterogeneity; values of 25%, 50% and 75% correspond to low, moderate and high heterogeneity, respectively.

The presence of publication bias for each endpoint was assessed by visual estimation with the use of contour-enhanced funnel plots [20]. The interpretation and meaning of contour-enhanced funnel plots have been reported elsewhere. To address potential sources of heterogeneity, we performed pre-specified random-effects meta-regression analyses assessing whether the following covariates in each group could be a treatment effect modifier with respect to the primary endpoint of overall RAO: age, female sex, smoking status, diabetes, weight, height, acute coronary syndrome on admission, and PCI following coronary angiography. For the primary endpoint, subgroup analyses were performed according to: (1) study design: randomized versus observational; (2) access sheath size; (3) dedicated versus nondedicated device for ulnar artery compression. For the primary and secondary outcomes for which at least moderate heterogeneity was found, a leave-one-out sensitivity analysis was performed by leaving out exactly one study to assess the consistency of the results. The statistical level of significance was two-tailed *p* < 0.05 for treatment effects. All analyses were performed using Stata/MP version 17 (StataCorp LLC, Lakeway, TX, USA) software.

A trial sequential analysis (TSA) was performed for the primary outcome to provide the optimum information size to reduce type I and II errors potentially arising from the meta-analytic model [21]. In the meta-analysis, optimum information size was defined as the number of patients or events from the included studies necessary to accept or reject the statistical hypothesis [22]. Relative risk reduction (RRR) was calculated from the analysis of low-bias risk trials by excluding high-risk-of-bias studies that could overestimate the intervention effect. The proportion in the control group in the cumulative meta-analysis, a 5% (α < 0.05; two-sided) risk of a type 1 error and 80% statistical power were chosen to calculate the optimum information size and the cumulative Z-curve’s eventual breach of relevant trial sequential monitoring boundaries. TSA software was used for the analysis (version 0.9 beta http://www.ctu.dk/tsa, accessed on 8 August 2022).

## 3. Results

**Figure 1:**
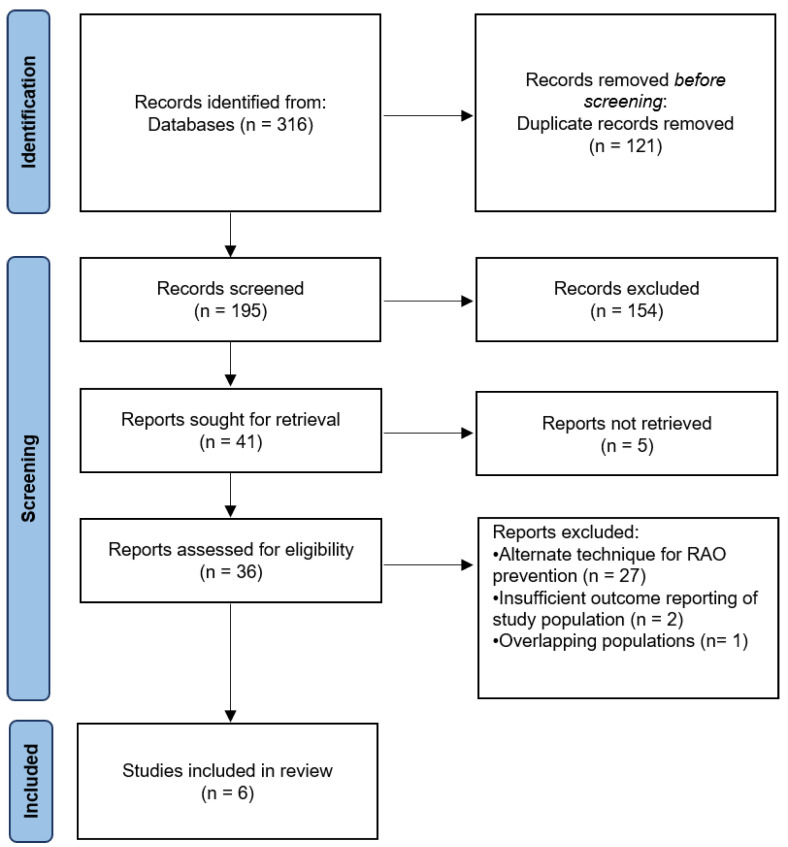
PRISMA flow diagram for search strategy. RAO, Radial artery occlusion.

The PRISMA flow diagram for study search and selection is shown in Figure 1. Of the 316 citations screened, a total of 6 studies, including 6793 patients, were identified and included in the final analysis [12,23,24,25,26,27]. Details on the search strategy are reported in the Supplementary Materials.

### 3.1. Study Characteristics and Bias Assessment

The trial and patient characteristics of the included studies are reported in Table 1 and Table S1.

The primary endpoint of overall RAO was reported in all studies. Coronary angiography alone was performed in one study [9], PCI following coronary angiography was performed in a variable proportion of patients ranging from 15.6% to 58.7% across 4 studies [23,25,26,27], while no information was available in one study [24]. Patients presenting with acute coronary syndrome were excluded in two studies [12,26], data regarding the clinical syndrome were reported in two studies [23,25], with the proportion of acute coronary syndrome ranging from 30.2% to 88.7%, and no information was available in 2 studies [24,27]. The best recommendations for the detection and prevention of RAO were followed in all included studies [10]. For instance, 5 French (Fr) access site sheath was used in 2 studies [12,24], 6 Fr access size sheath in 2 studies [26,27] and in the majority of cases (from 95.1% to 96.3%) in two studies [23,25]. Compression of the ulnar artery was achieved with a dedicated ulnar artery compression device comprising a double-balloon band system [23,24], a second radial band device adapted to the ulnar artery [26,27], or by compressing a cylindrical composite of gauze or the barrel of a plastic syringe at the Guyon’s canal with a Hemoband (Hemoband Corporation, Portland, Oregon) [12,25]. Transient ulnar artery compression (1 h) was performed in all studies but one [12], in which continuous compression following the radial artery compression time was applied.

Figure S1 summarizes the systematic bias assessment of the included RCT. Three out of 4 included studies showed a “low-risk” of bias [12,25,26], while there were “some concerns” for 1 study [24]. Table S2 summarizes the systematic bias assessment of the included observational studies. One study was judged as having a “high-risk” of bias [23], while one study raised “some concerns” [27].

### 3.2. Heterogeneity and Asymmetry

Significant heterogeneity was detected for the following endpoints: overall RAO, in-hospital RAO and unsuccessful patent hemostasis, as assessed by the Q chi-square test, and I^2^ was >50% (Table S3). Contour-enhanced funnel plots for all endpoints are reported in Figures S2–S7. Evidence for significant asymmetry was found for all endpoints, and the “missing studies” are expected to lie in areas of statistical significance so that the observed asymmetry is more likely to be due to factors other than publication bias based on statistical significance.

### 3.3. Outcomes

**Figure 2:**
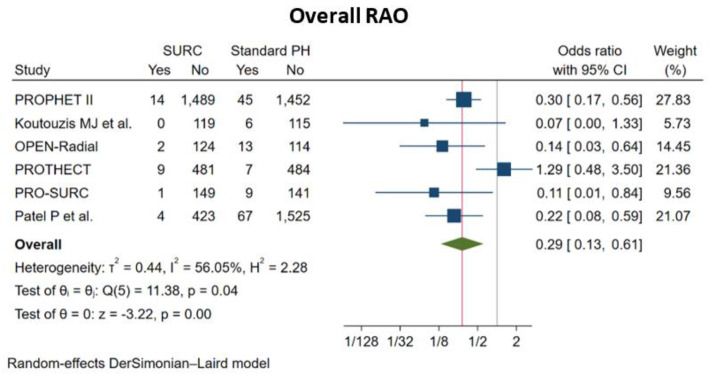
Pooled analysis of studies comparing simultaneous ipsilateral and ulnar artery compression (SURC) versus isolated radial artery compression. Forest plot reporting trial-specific and summary odds ratios (OR) with 95% confidence interval (CI) for the endpoint of overall radial artery occlusion (RAO) at the longest available follow-up. PH, Patent Hemostasis.

**Figure 3:**
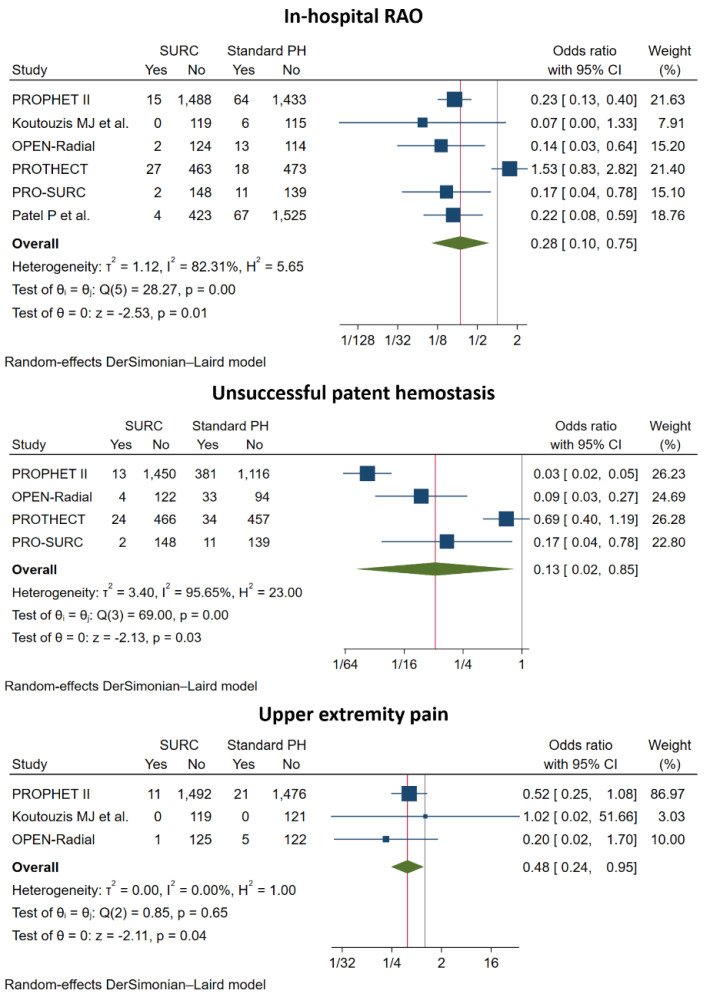
Pooled analysis of studies comparing simultaneous ipsilateral and ulnar artery compression versus isolated radial artery compression. Forest plot reporting trial-specific and summary odds ratios (OR) with 95% confidence interval (CI) for the endpoint of in-hospital radial artery occlusion (RAO), unsuccessful patent hemostasis (PH) and upper extremity pain.

**Figure 4:**
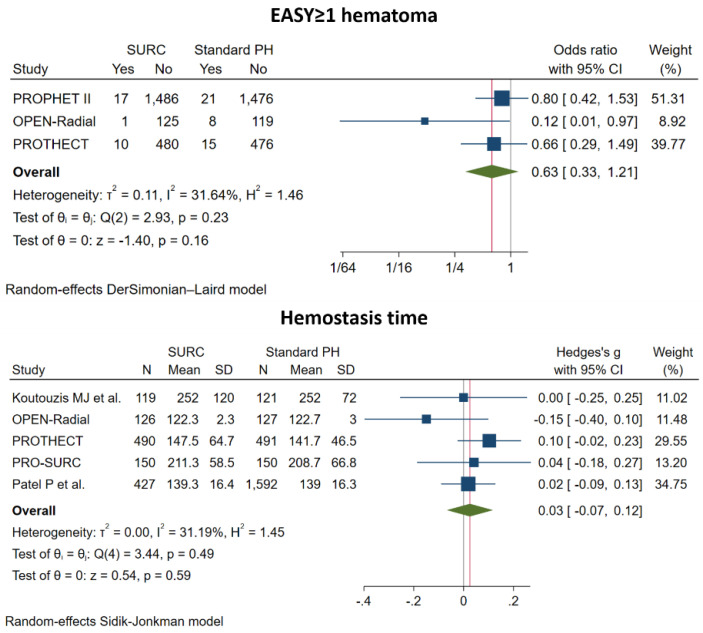
Pooled analysis of studies comparing simultaneous ipsilateral and ulnar artery compression versus isolated radial artery compression. Forest plot reporting trial-specific and summary odds ratio (OR) and standardized mean difference (SD) with 95% confidence interval (CI) for the endpoint of Early Discharge After Transradial Stenting of Coronary Arteries (EASY) ≥1 hematoma and hemostasis time. PH, Patent Hemostasis.

Radial and ipsilateral ulnar artery compression, compared with isolated radial artery compression, was associated with a significantly lower risk for overall RAO (OR: 0.29; 95% CI: 0.13 to 0.61; *p* < 0.001, NNTB = 38), in-hospital RAO (OR: 0.28; 95% CI: 0.10 to 0.75; *p* = 0.01, NNTB = 36), unsuccessful patent hemostasis (OR: 0.13; 95% CI: 0.02 to 0.85; *p* = 0.03, NNTB = 5) and reported upper extremity pain (OR: 0.48; 95% CI: 0.24 to 0.95; *p* = 0.04, NNTB = 124). There was no significant difference in the incidence of EASY ≥ 1 hematoma (OR: 0.63; 95% CI: 0.33 to 1.21; *p* = 0.16), hemostasis time (SMD: 0.03; 95% CI: −0.07 to 0.12; *p* = 0.59) (Figure 2, Figure 3 and Figure 4, Table S4).

### 3.4. Metaregression Analysis

Age (*p* = 0.85), female gender (*p* = 0.66), diabetes mellitus (*p* = 0.17), weight (*p* = 0.91), height (*p* = 0.73) and rate of PCI (*p* = 0.24) did not emerge as treatment effect modifiers for the primary endpoint, while smoking (*p* = 0.02) was associated with a reduced effect of SURC, compared with isolated radial artery compression, on the risk of overall RAO (Table S5, Figure S8).

### 3.5. Subgroup Analysis

Consistent results with the main analysis with respect to the comparison of SURC versus isolated radial artery compression on overall RAO were observed in the subgroup analysis according to: study design, randomized versus observational (p_interaction_ = 0.44) and to the access site sheath size, 6 Fr versus 5 Fr (p_interaction_ = 0.24) (Figures S9 and S10). However, when considering dedicated vs nondedicated devices for achieving ulnar artery compression, SURC with use of a nondedicated device was associated with a nonsignificant reduction in RAO (OR: 0.34; 95% CI: 0.11 to 1.07), whereas a dedicated two-balloon device maintained a significant reduction (OR: 0.19; 95% CI: 0.08 to 0.44). Nevertheless, there was no significant difference between groups (p_interaction_ = 0.41) (Figure S11).

### 3.6. Sensitivity Analysis

Similar results with respect to the comparison of SURC versus isolated radial artery compression on overall RAO were observed by leave-one-out analysis, while the risk of the secondary endpoint in-hospital RAO was no longer reduced after the removal of the PROPHET II trial [12] and the significantly lower rate of unsuccessful patent hemostasis seen with simultaneous compression was not obtained omitting two studies [24,26] (Figures S12–S14).

### 3.7. Trial Sequential Analysis

In TSA, the cumulative Z-curve exceeded both the conventional and TSA monitoring boundaries for the primary outcome. The pooled sample size exceeded the calculated optimum sample size, indicating that conclusions on the primary outcome were robust and were hardly modified with additional related trials (Figure S15).

## 4. Discussion

This meta-analysis, including 6793 patients from 4 RCTs and two observational studies, provides a comprehensive evaluation of the effects of SURC versus isolated radial artery compression after conventional radial access for coronary angiography and/or intervention. The main study findings are as follows: (1) SURC technique as compared to isolated radial artery compression is associated with a 71% relative risk reduction in overall RAO and a 72% relative risk reduction in in-hospital RAO, with a robust absolute treatment benefit as evidenced by an NNTB of 38 and 36, respectively; (2) the use of SURC was associated with successful patent hemostasis in 98% of patients versus 80% of patients undergoing isolated radial artery compression with the patent hemostasis protocol; (3) there was a lower risk of reported upper extremity pain during hemostasis using SURC technique; (4) the augmentation of radial blood flow through ulnar artery compression does not increase the risk of access site hematoma and does not extend hemostasis time.

Prevention of RAO after TRA is a crucial aspect to ensure viable access for future procedures, an important consideration for patients with coronary artery disease, which often require repeated percutaneous access. Many strategies, both pharmacological and non-pharmacological, have been employed to prevent RAO [10]. The benefits of patent hemostasis on the prevention of forearm RAO have long been observed. The present study focuses on a non-pharmacological iteration of patent hemostasis using SURC. The findings of this meta-analysis were robustly in favor of this new hemostasis protocol to reduce the risk of overall and in-hospital RAO. Analysis of absolute treatment effects showed that the benefits are of clinical relevance given the NNTB of 36 and 38 for overall and in-hospital RAO corresponding to 26 and 27 events avoided per 1000 patients treated. The underlying mechanism supporting these results are based on increased flow-mediated vasodilation of the radial artery provided by ulnar artery compression, which provides supportive circumferential forces against continuous radial mechanical compression. Indeed, radial artery patency mechanically relies on the centrifugal forces driven by intraluminal pressure ejecting blood through the artery together with its circumferential stress on one side, and the forces of hemostatic compression on the other. These forces may vary in the time interval between the end of the procedure (e.g., from patient relaxation and blood pressure drop) and the start of deflation of the hemostasis device, while the extrinsic compression force remains constant, which can ultimately result in a dominating mechanical compressive force and therefore radial lumen compromise and flow cessation.

Despite its efficacy in lowering RAO, the patent hemostasis protocol has received limited adoption in cardiac catheterization laboratories worldwide, likely due to the frequent monitoring required and the scarcity of skilled manpower. In our study, the SURC technique was associated with 87% relative risk reduction of unsuccessful patent hemostasis with potential logistical advantages that this innovative hemostasis technique poses on post-procedure patient management. By compressing the ulnar artery, it is possible to continuously monitor for RAO during ulnar compression by the continuous application of the reverse Barbeau test (oximetry plethysmographic monitoring). Automation by using alarms if no plethysmographic signal is detected also allows for the reduction in point-of-care post-procedure evaluations to reassure patent hemostasis.

Furthermore, TRA is often associated with post-procedural arm pain and patient’s discomfort. Prolonged hemostatic compression seems to represent the most important factor associated with reported pain, and could be associated with increased radial nerve nociceptive fibers activation. In addition, RAO after TRA seems to be also associated with post-procedural upper extremity pain. Indeed Dharma et al. found that among 1706 patients, RAO was significantly associated with increased incidence of post-procedural pain after TRA, although in absence of limb ischemia [28]. Possible mechanism of non-ischemic limb pain includes thrombus dependent arteritis and radial nerve irritation. This would partly explain the reduced incidence of upper limb pain with simultaneous ulnar compression in the present meta-analysis.

Two studies used dedicated devices designed with two-bladder compression to supply ulnar artery occlusion and patent hemostasis of the radial artery (Vasoband, Vasoinnovations Inc., South Pasadena, CA, USA) [23,24]. At subgroup analysis, these dedicated devices showed a trend towards an even more profound reduced risk of RAO compared to the use of nondedicated devices to achieve hemostasis compressing both ulnar and radial artery simultaneously, although differences between subgroups were not significant (Figure S13). This could be attributed to the reduced risk of inadvertent excess radial compression during ulnar compression by using a single-band two-bladder system. Head-to-head comparison of techniques based on simultaneous compression of the ulnar and radial arteries to achieve hemostasis after TRA are warranted to elucidate any advantages of dedicated devices.

A relevant procedural difference to consider between the included studies is represented by the differences in sheath size, which in itself, is an important predictor of RAO [1,29]. No difference was found between studies using 5-Fr versus 6-Fr access sheath size. One study included patients undergoing TRA with 7 Fr sheaths in the experimental arm. Despite the larger introducer size, the investigators reported a RAO incidence similar to that observed with 6 Fr systems (0.9% vs. 1.0%, *p* = 0.9) [23]. A RCT comparing thin-walled radial sheath to standard sheath for large-bore access, failed to demonstrate a significant reduction in the incidence of RAO within 24 h following complex PCI. [30] Therefore, one subset of patients in which this hemostasis technique could be implemented preferentially over standard hemostasis in the future is represented by patients undergoing complex PCI via TRA, requiring larger sheath sizes, thus implying a greater risk of RAO. Importantly, the study by Patel et al. [20], was observational in nature, and randomized data would be needed to adequately assess the effectiveness of the SURC technique in preventing RAO after TRA for complex PCI using large-bore access.

To our knowledge, this is the first published meta-analysis comparing the efficacy of SURC technique versus isolated radial artery compression after TRA. These data provide a supportive cornerstone for the widespread adoption of this simple yet innovative hemostasis technique and the development of other dedicated devices. Future studies are warranted to determine which of the approach described so far [31,32] is the safest and most beneficial for the prevention of RAO.

## 5. Limitations

Additionally, caution is needed in interpreting our results owing to some limitations. First, the inclusion of observation data in the meta-analysis inevitably increases the risk of bias due to unmeasured confounders. However, subgroup analysis of RCTs and observational studies did not reveal any difference between the two groups (p_interaction_ = 0.44). Second, the absence of patient-level data prevents assessment of the impact of many baseline characteristics on the efficacy and safety outcomes at the patient level. Lastly, different modalities were used to achieve hemostasis across the included studies; thus, the results should not be interpreted as supporting one modality over another. Future studies should be designed to provide insights into this field.

## 6. Conclusions

In addition to patent hemostasis of the radial artery after TRA, ulnar artery compression is associated with reduced risk of RAO, unsuccessful patent hemostasis and upper extremity pain, without a trade-off in access-site complications. Thus, these data support the widespread adoption of this simple and effective technique for patients undergoing coronary angiography and/or intervention via TRA.

## Figures and Tables

**Table 1:** Main trial and patient characteristics of included studies.

Study	PROPHET II [12]	Koutouzis MJ et al [27]	OPEN-Radial [24]	PROTHECT [25]	PRO-SURC [26]	Patel P et al [23]
Country, date	India, Czech Republic, 2016	Greece, 2016	India, 2020	Mexico, 2022	Egypt, 2022	India, 2022
Study design	RCT, open-label	Observational, prospective	RCT, open-label	RCT, open-label	RCT, open-label	Observational, retrospective
Number of patients	3000	240	253	981	300	2019
Access sheath size	11-cm long 5-F hydrophilic introducer sheath (Terumo Interventional Systems, Tokyo, Japan).	11-cm long 6 Fr hydrophilic Arrow sheaths (Teleflex, Limerick, PA, USA).	7-cm long 5 Fr hydrophilic-coated, Radifocus introducer sheath (Terumo Interventional Systems, Tokyo, Japan)	6 Fr radial sheath, 464 (94.5) SURC:6 Fr radial sheath, 469 (95.7)	6 Fr radial sheath.	6 Fr or 7 Fr slender introducer sheath (Terumo Interventional Systems) selected based on operator’s discretion.
Hemostasis system	Isolated radial artery compression: TR band (Terumo Interventional Systems, Tokyo, Japan). SURC: radial artery compression with TR band (Terumo Interventional Systems). The ipsilateral ulnar artery was compressed at the Guyon’s canal by placing a cylindrical composite made by wrapping 4 inch × 4 inch gauze around a 1-inch plastic needle cap, or the barrel of a 3 mL plastic syringe, and compressing it using a circumferentially applied Hemoband (Hemoband Corporation, Portland, Oregon).	Isolated radial artery compression: Tourniquet screw-down pressure plate hemostatic device (KDL, type ZXD II-22; Shanghai Kindly Enterprise Development Group Co, Shangai, China). SURC: Tourniquet screw-down pressure plate hemostatic device (KDL, type ZXD II-22; Shanghai Kindly Enterprise Development Group Co, Shangai, China).1-hour ipsilateral ulnar compression with another of the same device.	Isolated radial artery compression: TR band (Terumo Interventional Systems, Tokyo, Japan).SURC: Two-bladder Vasoband (Vasoinnovations Inc.) plus ipsilateral ulnar compression (dedicated device).	Isolated radial artery compression: TR band (Terumo Interventional Systems). SURC: TR band (Terumo Interventional Systems, Tokyo, Japan) Ipsilateral ulnar artery compression was achieved by placing 4-inch × 4- inch cylindrical-shaped gauze that was compressed against the distal third of the ulnar artery by a 14- inch standard-length Hemoband device (Hemoband Corporation, Portland, Oregon).	Isolated radial artery compression: Balloon inflatable radial hemostatic band (TR band, Terumo Interventional System, Tokyo, Japan). Balloon inflatable radial hemostatic band (TR band). SURC: The ipsilateral ulnar artery was compressed by placing a second inflatable band proximal to the radial compression band in order to increase the velocity of blood flow into the radial artery.	Isolated radial artery compression: One-bladder TR band (Terumo Interventional Systems, Tokyo, Japan). SURC: Two-bladder Vasoband (Vasoinnovations Inc., South Pasadena, CA, USA) with ipsilateral ulnar compression (dedicated device).
Timing and modality of RAO assessment	At the time of removal of the radial compression band, 24 h and 30 days following the procedure by pulse plethysmography or oximetry. In patients where compression of both radial and ulnar arteries did not result in total loss of plethysmographic signal, and in those where RAO was detected by digital plethysmography, duplex ultrasonography was performed to confirm patency status.	Within 1 h after hemostasis device removal by radial artery pulsation and when needed by Duplex US.	At the time of discharge, at a minimum of 1 hour after the removal of the hemostatic compression device by plethysmographic technique. All equivocal plethysmographic tests were evaluated by performing radial artery duplex Doppler US.	At 24 hours after the removal of the introducer sheath by oximetry plethysmography. In the presence of RAO by plethysmography, Doppler ultrasound) was performed to corroborate the occlusion. Patients with Doppler US criteria for RAO were evaluated with a repeat Doppler US study at 30 days.	Duplex US assessment was performed at 1-h post-TR band removal and after one month.	At 24 h after the procedure by US.

Abbreviations: RAO, radial artery occlusion; RCT, randomized controlled trial, SURC, simultaneous ipsilateral ulnar and radial artery compression; TR, transradial; US, ultrasound.

## Data Availability

Not applicable.

## Supplementary Materials

The following supporting information can be downloaded at: https://www.mdpi.com/article/10.3390/jcm11237013/s1, Table S1: Additional main trial and patient characteristics of included studies; Table S2: Risk of Bias assessment—Observational studies. Risk of Bias In Non-randomized Studies of Interventions assessment Tool from Cochrane handbook (ROBINS-I) for the outcome of overall radial artery occlusion; Table S3: Heterogeneity measures in the overall population; Table S4: Absolute treatment effect measures in the overall population: distal vs conventional radial artery; Table S5: Random-effects meta-regression analysis assessing the effect of covariates on the relative risk of the primary endpoint of overall RAO; Figure S1: Risk of bias summary: judgements about each risk of bias item for each randomized study; Figure S2: Contour-enhanced funnel plot for the pooled analysis of studies comparing simultaneous ulnar and radial artery compression vs isolated radial artery compression for the endpoint of overall RAO; Figure S3: Contour-enhanced funnel plot for the pooled analysis of studies comparing simultaneous ulnar and radial artery compression vs isolated radial artery compression for the endpoint of in-hospital RAO; Figure S4: Contour-enhanced funnel plot for the pooled analysis of studies comparing simultaneous ulnar and radial artery compression vs isolated radial artery compression for the endpoint of unsuccessful patent hemostasis; Figure S5: Contour-enhanced funnel plot for the pooled analysis of studies comparing simultaneous ulnar and radial artery compression vs isolated radial artery compression for the endpoint of upper extremity pain; Figure S6: Contour-enhanced funnel plot for the pooled analysis of studies comparing simultaneous ulnar and radial artery compression vs isolated radial artery compression for the endpoint of EASY ≥ 1 hematoma; Figure S7: Contour-enhanced funnel plot for the pooled analysis of studies comparing simultaneous ulnar and radial artery compression vs isolated radial artery compression for the endpoint of hemostasis time; Figure S8: Random-effects meta-regression showing the effect of smoking on the odds ratio of the primary endpoint overall RAO of simultaneous ipsilateral ulnar and radial artery compression versus isolated radial artery compression; Figure S9: Pooled analysis of studies comparing simultaneous ipsilateral ulnar and radial artery compression versus isolated radial artery compression. Forest plot reporting trial-specific and summary odds ratios with 95% confidence interval (CI) for the endpoint of overall RAO according to study design; Figure S10: Pooled analysis of studies comparing simultaneous ipsilateral ulnar and radial artery compression versus isolated radial artery compression. Forest plot reporting trial-specific and summary odds ratios with 95% confidence interval (CI) for the endpoint of overall RAO according to the access sheath size; Figure S11: Pooled analysis of studies comparing simultaneous ipsilateral ulnar and radial artery compression versus isolated radial artery compression. Forest plot reporting trial-specific and summary odds ratios with 95% confidence interval (CI) for the endpoint of overall RAO according to the use of dedicated versus nondedicated ulnar artery compression devices; Figure S12: Leave-one out sensitivity analysis for the primary endpoint of overall RAO. Forest plot of summary odds ratios (RR) with 95% confidence interval (CI) after removing one study in turn; Figure S13: Leave-one out sensitivity analysis for the secondary endpoint of in-hospital RAO. Forest plot of summary odds ratios (OR) with 95% confidence interval (CI) after removing one study in turn; Figure S14: Leave-one out sensitivity analysis for the secondary endpoint of unsuccessful patent hemostasis. Forest plot of summary odds ratios (OR) with 95% confidence interval (CI) after removing one study in turn; Figure S15: Trial sequential analysis of the primary endpoint using random-effects meta-analysis, based on low-bias risk computed relative risk reduction, a control event incidence with alpha 5% and statistical power of 80%.
