# Supplementary material for: Simultaneous Radial and Ipsilateral Ulnar Artery Compression versus Isolated Radial Artery Compression after Conventional Radial Access for Coronary Angiography and/or Intervention: A Systematic Review and Meta-Analysis
Source: J Clin Med. 2022 Nov 27;11(23):7013. doi: 10.3390/jcm11237013 (PMC9739321; doi:10.3390/jcm11237013)

Supplemental Material includes:

Search strategy

Figure legends for Figures from 1 to 15

Table S1 to S5

Figures S1 to S15

This supplementary material has been provided by the authors to give readers additional information about their work.

Database Search Results

Database	Search Strategy	Search Results
PubMed/MEDLINE	("radial" [tiab] AND ("occlusion"[tiab] OR "thrombosis"[tiab] OR "RAO"[tiab])) AND ("ulnar" [tiab] OR "ipsilateral"[tiab]) AND ("hemosta*" [tiab] OR "compress*" [tiab])	54
EMBASE	(('radial':ab) AND ('occlusion':ab OR 'thrombosis':ab OR 'RAO':ab)) AND ('ulnar':ab OR 'ipsilateral':ab) AND ('hemosta*':ab OR 'compress*':ab)	106
SCOPUS	TITLE-ABS-KEY (("radial" AND ("occlusion" OR "thrombosis" OR "RAO")) AND ("ulnar" OR "ipsilateral") AND ("hemosta*" OR "compress*"))	129
Cochrane	("radial" AND ("occlusion" OR "thrombosis" OR "RAO")) AND ("ulnar" OR "ipsilateral") AND ("hemosta*" OR "compress*")	27

EMBASE = Excerpta Medica Database; MEDLINE = Medical Literature Analysis and Retrieval System Online.

Figure legends

Figure S1: Risk of bias summary: judgements about each risk of bias item for each randomized study.

Figure S2: Contour-enhanced funnel plot for the pooled analysis of studies comparing simultaneous ulnar and radial artery compression vs isolated radial artery compression for the endpoint of overall RAO.

Figure S3: Contour-enhanced funnel plot for the pooled analysis of studies comparing simultaneous ulnar and radial artery compression vs isolated radial artery compression for the endpoint of in-hospital RAO.

Figure S4: Contour-enhanced funnel plot for the pooled analysis of studies comparing simultaneous ulnar and radial artery compression vs isolated radial artery compression for the endpoint of unsuccessful patent hemostasis.

Figure S5: Contour-enhanced funnel plot for the pooled analysis of studies comparing simultaneous ulnar and radial artery compression vs isolated radial artery compression for the endpoint of upper extremity pain.

Figure S6: Contour-enhanced funnel plot for the pooled analysis of studies comparing simultaneous ulnar and radial artery compression vs isolated radial artery compression for the endpoint of EASY≥I hematoma.

Figure S7: Contour-enhanced funnel plot for the pooled analysis of studies comparing simultaneous ulnar and radial artery compression vs isolated radial artery compression for the endpoint of hemostasis time.

Figure S8: Random-effects meta-regression showing the effect of smoking on the odds ratio of the primary endpoint overall RAO of simultaneous ipsilateral ulnar and radial artery compression versus isolated radial artery compression.

Figure S9: Pooled analysis of studies comparing simultaneous ipsilateral ulnar and radial artery compression versus isolated radial artery compression. Forest plot reporting trial-specific and summary odds ratios with 95% confidence interval (CI) for the endpoint of overall RAO according to study design.

Figure S10: Pooled analysis of studies comparing simultaneous ipsilateral ulnar and radial artery compression versus isolated radial artery compression. Forest plot reporting trial-specific and summary odds ratios with 95% confidence interval (CI) for the endpoint of overall RAO according to the access sheath size.

Figure S11: Pooled analysis of studies comparing simultaneous ipsilateral ulnar and radial artery compression versus isolated radial artery compression. Forest plot reporting trial-specific and summary odds ratios with 95% confidence interval (CI) for the endpoint of overall RAO according to the use of dedicated versus non-dedicated ulnar artery compression devices.

Figure S12: Leave-one out sensitivity analysis for the primary endpoint of overall RAO. Forest plot of summary odds ratios (RR) with 95% confidence interval (CI) after removing one study in turn.

Figure S13: Leave-one out sensitivity analysis for the secondary endpoint of in-hospital RAO. Forest plot of summary odds ratios (OR) with 95% confidence interval (CI) after removing one study in turn.

Figure S14: Leave-one out sensitivity analysis for the secondary endpoint of unsuccessful patent hemostasis. Forest plot of summary odds ratios (OR) with 95% confidence interval (CI) after removing one study in turn.

Figure S15: Trial sequential analysis of the primary endpoint using random-effects meta-analysis, based on low-bias risk computed relative risk reduction, a control event incidence with alpha 5% and statistical power of 80%.

[illegible]

or cardiac catheterization using	Patients 18 years old or older in whom a radial access was successfully obtained to perform a coronary diagnostic or therapeutic procedure.		Consecutive patients referred for elective diagnostic CAG or PCI via TRA.		Patients referred for CAG or PCI between January 1, 2020, and December 31, 2021.	
history of previous ipsilateral who received warfarin or on agulant compounds; 3)patients nuous intravenous heparin indication; 4) patients with ysmography waveform when ulnar artery were occlusively estive of presumed interosseous digital plethysmography ents with absence of palpable patients who did not provide .	Patients who refused to participate; those who had a recent transradial procedure in the same radial artery (< 1 month), a failed transradial access, or crossover of the initial access; and patients in cardiogenic shock were excluded.		Pre-procedural duplex detection of radial artery anomalies as hypoplasia, tortuosity and severe calcification or radial artery diameter < 2 mm; 2) Under anticoagulant therapy; 3) Presented with ACS; 4) Failure to cannulate the radial artery, and 5) Failure of navigation due to extreme tortuosity or vascular loops above brachial bifurcation.		Long - term oral anticoagulation, previous ipsilateral radial artery access, postprocedural systemic anticoagulation, hemodynamic instability requiring vasopressors in the periprocedural period.	
cktail containing 200 μg of d 2.5 mg of verapamil was a-arterially via the introducer, us of UFH was administered r placement of the sheath.	A cocktail of 5,000 IU of UFH, 2.5 mg verapamil, and 200 mg nitroglycerin was administered through the sheath. In PCI cases, an additional dose of UFH was administered to complete a dose of 100 IU/kg. The dose of UFH was adjusted according to the ACT measured 1 hour after the start of the procedure.		200 μg of nitroglycerin given through the introducer sheath, verapamil at the operator's discretion. 5000 IU, UFH given intra-arterially through the sheath side arm to all patients. If PCI was decided by the operator, more UFH was given (total of 100 IU/kg patient weight).		200 μg of nitroglycerin with either diltiazem 5 or 2.5 mg of Verapamil in a 10 ml 0.9% saline solution. UFH, 50 IU/kg of body weight was administered intravenously after placement of the introducer sheath for CAG and 100 IU/kg for planned PCI. For ad hoc PCI, additional 50 IU/kg of UFH was given. Further heparin boluses were administered based on ACT at the operator's discretion.	
120<br>Transient ulnar artery compression (1 hour).	120	120<br>Transient ulnar artery compression (1 hour).	Gradual device deflation was performed after 2 hours for diagnostic CAG and 4 hours for PCI<br>Transient ulnar artery compression (1 hour).		120 minutes. For patients receiving Vasoband, the ulnar bladder was deflated at 60 minutes.	
Yes	Yes	Yes	Yes	Yes	Yes	
NA	245 (49.9)	229 (46.8)	91 (60.7)	85 (56,7)	262 (16)	54(13)
NA	436 (88.8)	434 (88.6)	0	0	478 (30)	132 (31)
59 (11)	60 (11.2)	61 (10.8)	57,4 (9,4)	55 (8)	63 (11.9)	66.3 (11.2)
93 (73.8)	354 (72)	354 (72.2)	128 (85,3)	126 (84)	1114 (70)	312 (73)
164.7 (8.2)	NA	NA	NA	NA	167.7 (11.1)	170.3 (11.2)
72 (11.2)	NA	NA	NA	NA	76 (17.1)	84 (21.6)
79 (62.7)	257 (52.3)	270 (55.1)	59 (39.3)	61 (40.7)	1067 (67)	299 (70)
NA	142 (28.9)	142 (29)	45 (30)	55 (36.7)	NA	NA
46 (36.5)	200 (40.7)	200 (40.8)	63 (42)	46 (30.7)	653 (41)	171(40)
NA	32 (6.5)	34 (6.9)	NA	NA	NA	NA
0	47 (9.6)	56 (11.4)	0	0	0	0

Table S2: Risk of Bias assessment – Observational studies. Risk of Bias In Non-randomized Studies of Interventions assessment Tool from Cochrane handbook (ROBINS-I) for the outcome of overall radial artery occlusion.

**Study**		**Pre-Intervention**		**At Intervention**	**Post-intervention**				**Overall risk of bias**
Study	***Year***	***Bias due to confounding***	***Bias in selection of participants into the study***	***Bias in classification of interventions***	***Bias due to deviations from intended interventions***	***Bias due to missing data***	***Bias in measurement of outcomes***	***Bias in selection of the reported result***	***Low/ moderate/ high***
Koutouzis et al.	2016	⚠	✓	✓	✓	✓	✓	✓	⚠
Patel et al.	2022	✗	⚠	✓	✓	✓	✓	✓	✗

✓ = low risk; ⚠ = moderate risk; ✗ = high risk

Table S3: Heterogeneity measures in the overall population.

	I^2	Tau^2	Q (Heterogeneity)	P value
Overall RAO	56.1	0.44	11.4	0.04
In-hospital RAO	82.3	1.12	28.3	<0.001
Unsuccessful patent hemostasis	95.6	3.40	69.0	<0.001
Hematoma EASY≥I	31.6	0.11	2.9	0.23
Hemostasis time	31.2	0.00	3.4	0.49
Upper extremity pain	0.0	0.00	0.85	0.65

EASY: early discharge after transradial stenting of coronary arteries grading; CI: confidence interval; RAO: radial artery occlusion;
*Values of I^2 are percentages.

Table S4: Absolute treatment effect measures in the overall population: distal vs conventional radial artery.

	Absolute risk difference	NNTB/NNTH	95% CI NNTB/NNTH	No of events avoided/caused per 1000 (95% CI)
Overall RAO	0.0263	38	30 to 52	Avoided 26 (19-33)
In-hospital RAO	0.02793	36	28 to 50	Avoided 27 (20-36)
Unsuccessful patent hemostasis	0.18336	5	5 to 6	Avoided 200 (167-200)
Hematoma EASY≥II	0.0076	131	NNTB 64 to ∞ to NNTH 5501	Avoided 8 (16 to “0” to caused 0.2)
Upper extremity pain	0.00803	124	67 to 864	Avoided 8 (2-15)

EASY: early discharge after transradial stenting of coronary arteries grading; CI: confidence interval; NNTB: number needed to treat to benefit; NNTH: number needed to treat to harm, RAO: radial artery occlusion.

Table S5: Random-effects meta-regression analysis assessing the effect of covariates on the relative risk of the primary endpoint of overall RAO.

	coefficient	95% CI	P value
Age	-3.1	-22.0 to 15.8	0.85
Female sex	-1.6	-3.1 to -0.1	0.66
Smoking	-4.04	-6.7 to 1.4	0.02
Diabetes mellitus	-1.7	-3.2 to -0.1	0.169
Weight	-5.4	-15.4 to 14.3	0.91
Height	7.6	-42.4 to 57.6	0.73
PCI	-1.8	-3.3 to -3.8	0.24

ACS: acute coronary syndrome; BMI: body mass index; PCI: percutaneous coronary intervention.

Figure S1: Risk of bias summary: judgements about each risk of bias item for each randomized study.

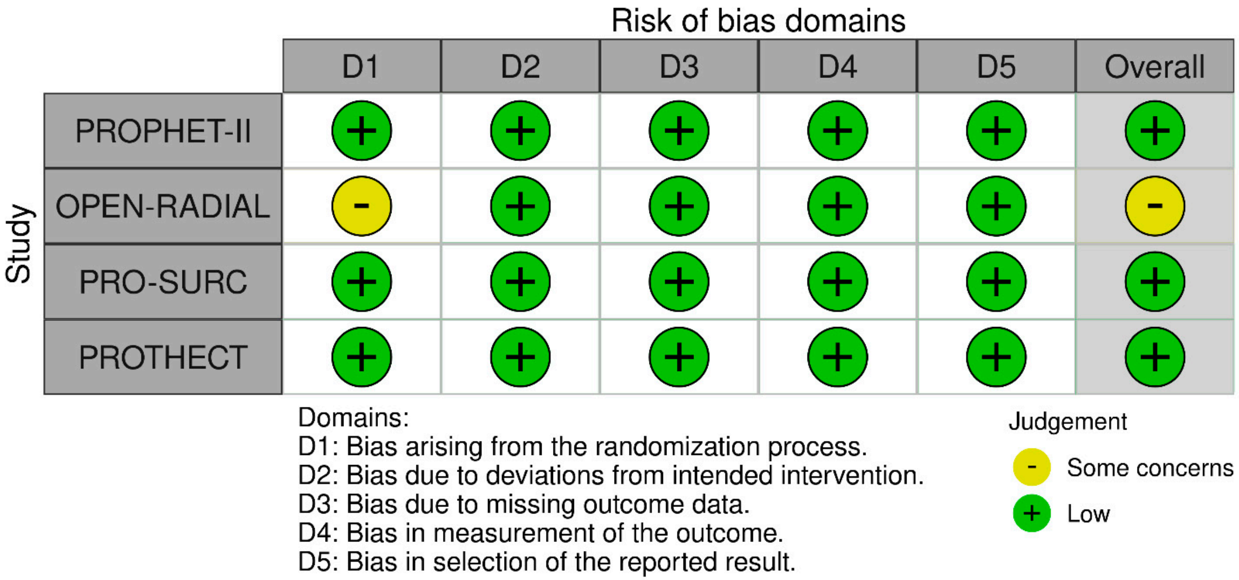

Figure S2: Contour-enhanced funnel plot for the pooled analysis of studies comparing simultaneous ulnar and radial artery compression vs isolated radial artery compression for the endpoint of overall RAO.

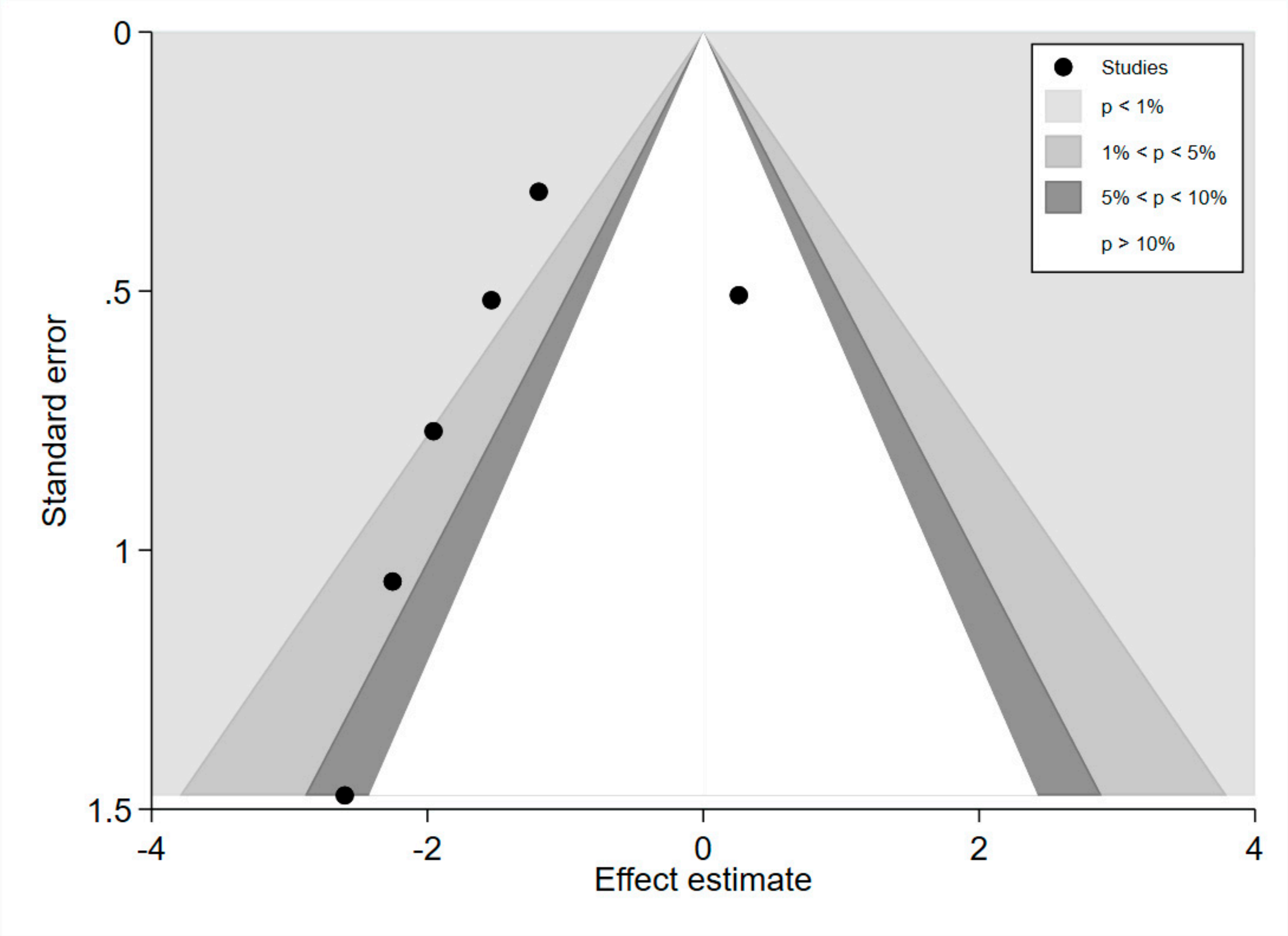

Figure S3: Contour-enhanced funnel plot for the pooled analysis of studies comparing simultaneous ulnar and radial artery compression vs isolated radial artery compression for the endpoint of in-hospital RAO.

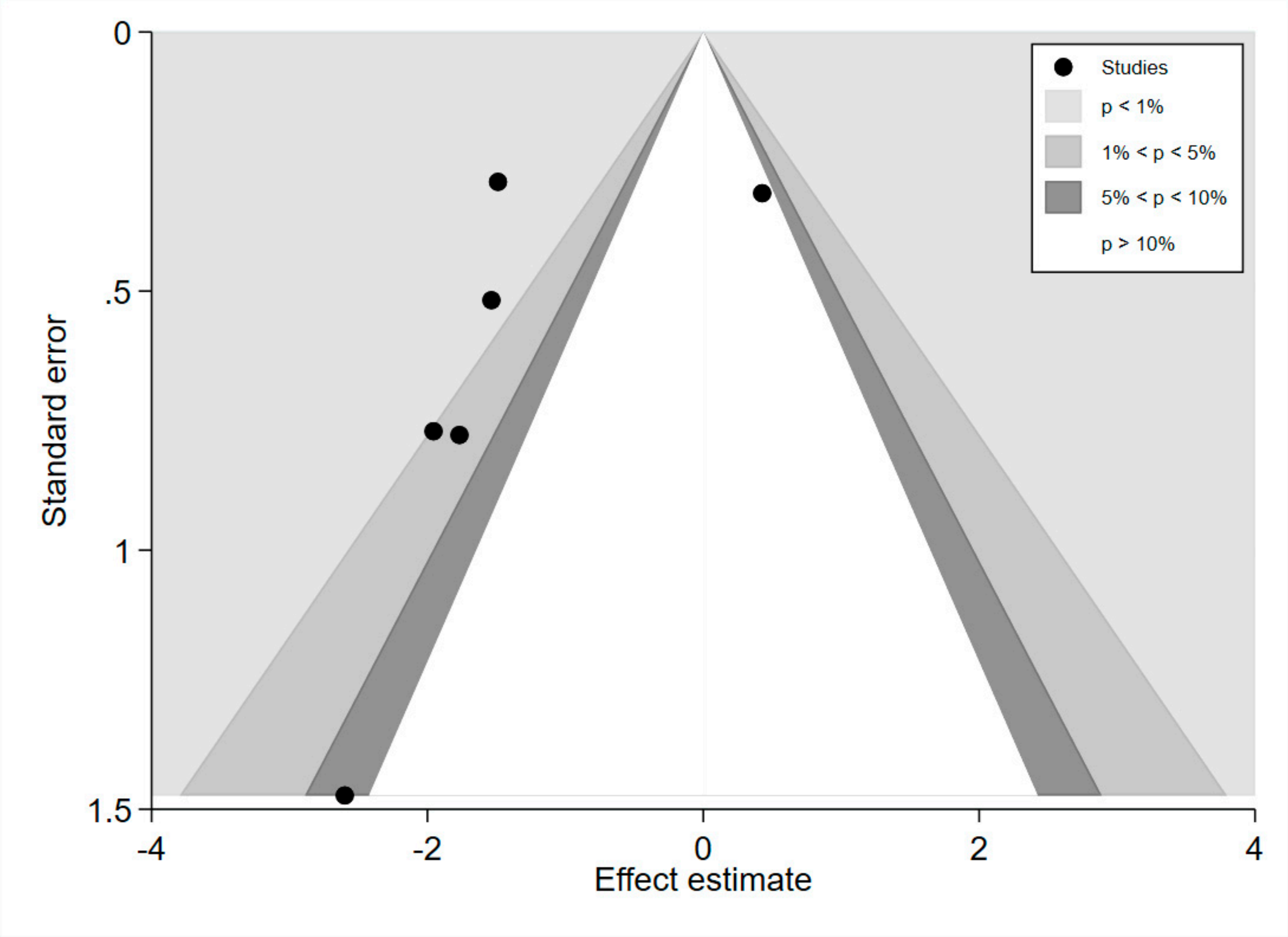

Figure S4: Contour-enhanced funnel plot for the pooled analysis of studies comparing simultaneous ulnar and radial artery compression vs isolated radial artery compression for the endpoint of unsuccessful patent hemostasis.

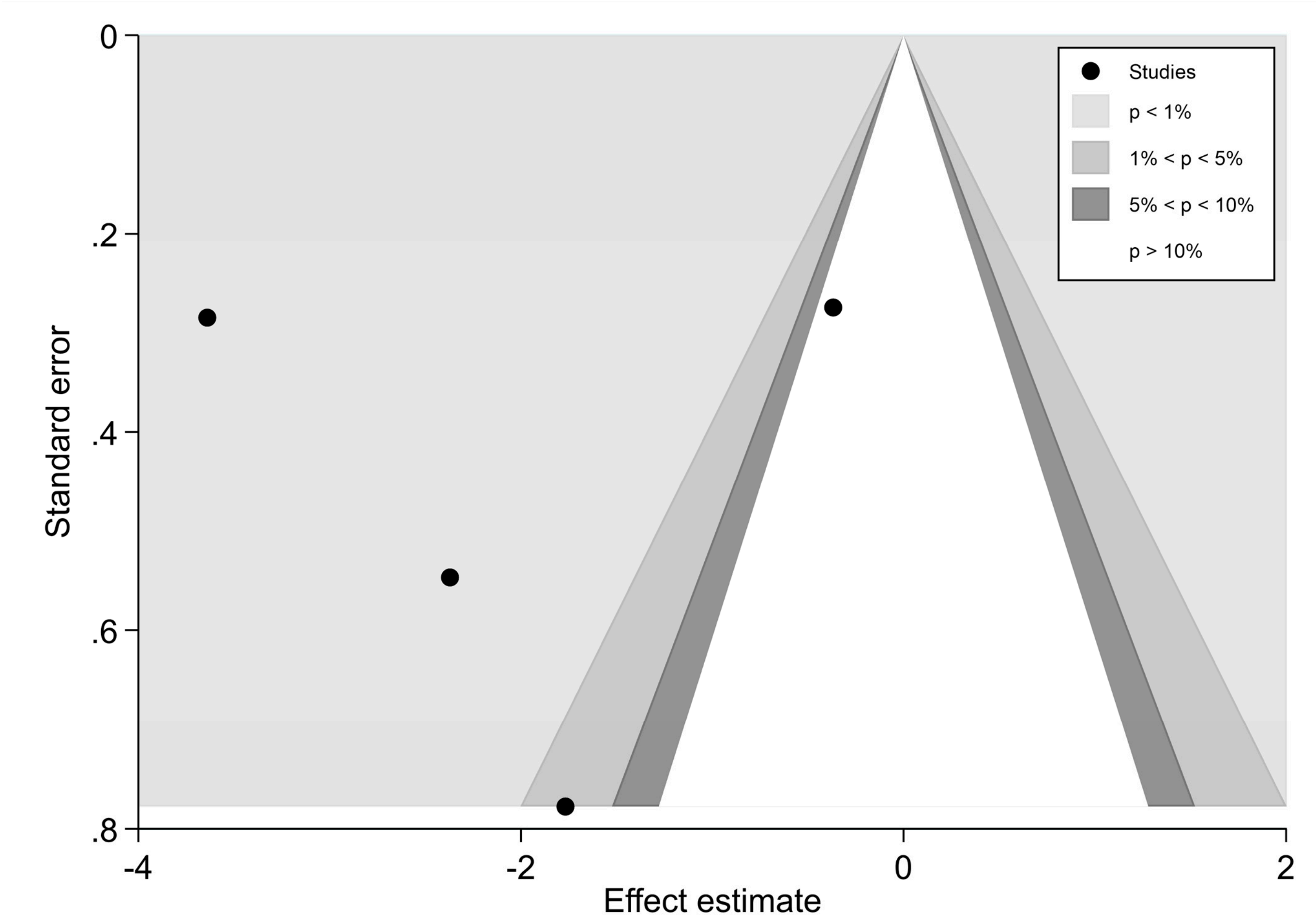

Figure S5: Contour-enhanced funnel plot for the pooled analysis of studies comparing simultaneous ulnar and radial artery compression vs isolated radial artery compression for the endpoint of upper extremity pain.

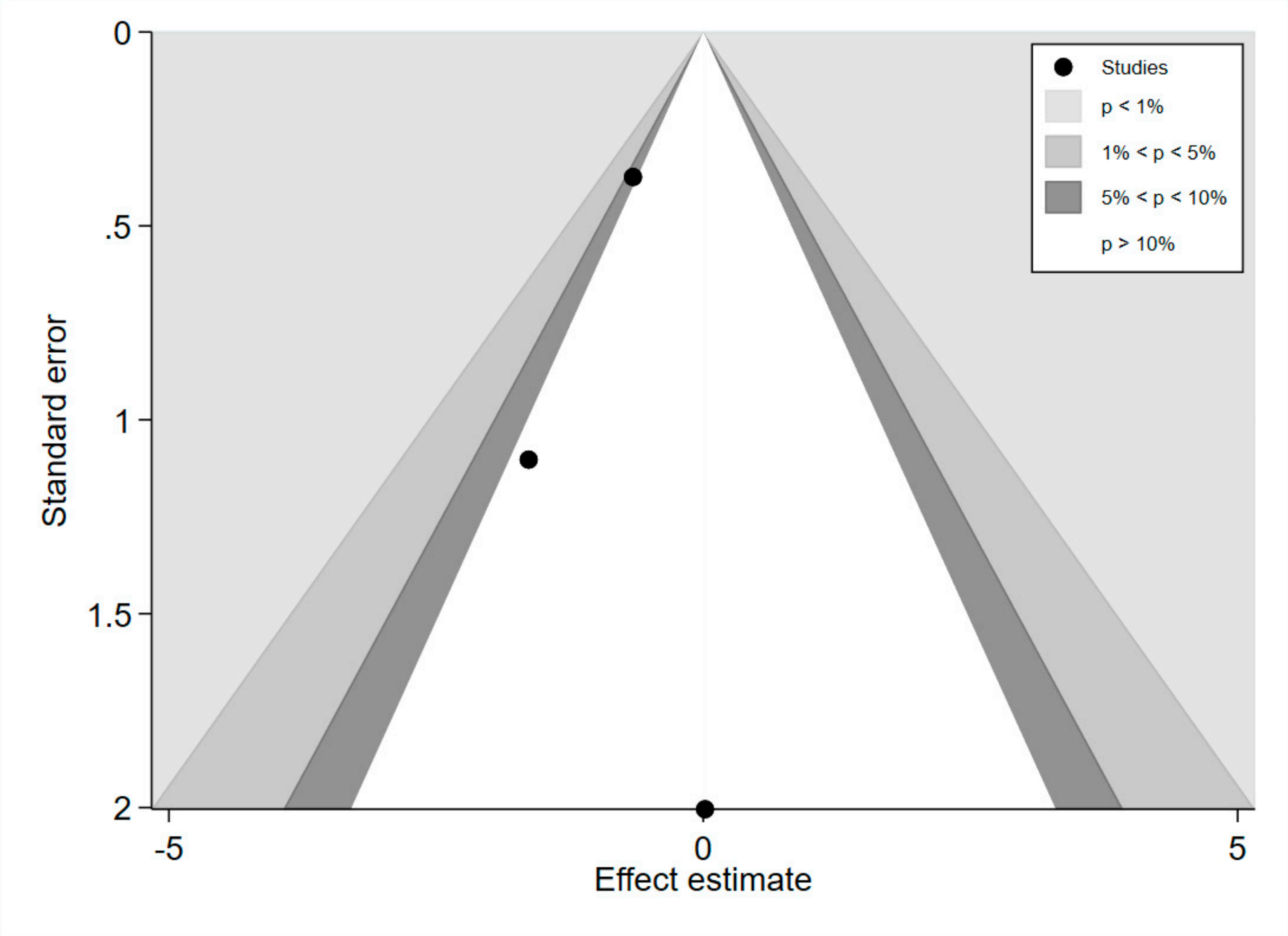

Figure S6: Contour-enhanced funnel plot for the pooled analysis of studies comparing simultaneous ulnar and radial artery compression vs isolated radial artery compression for the endpoint of EASY≥1 hematoma.

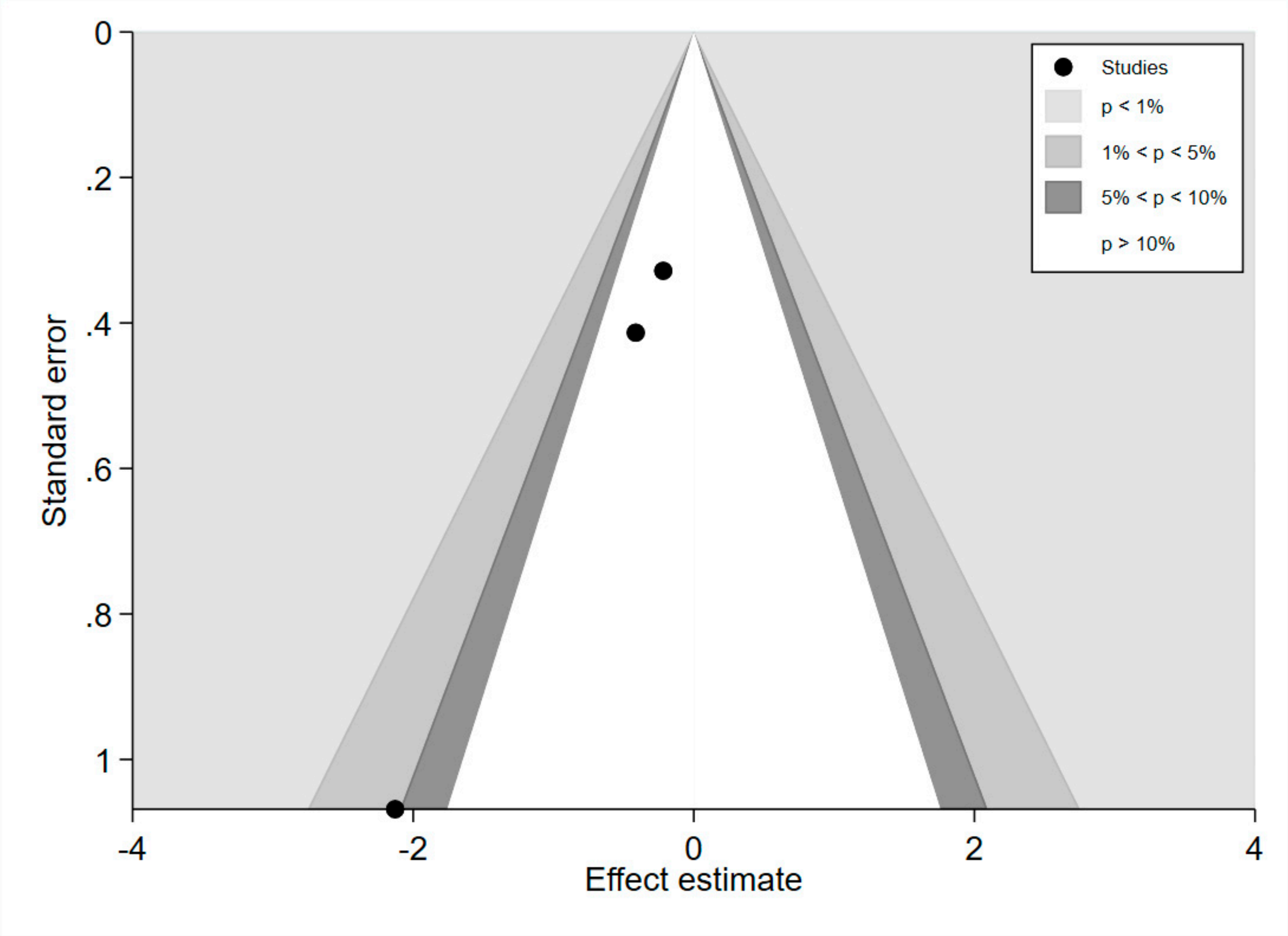

Figure S7: Contour-enhanced funnel plot for the pooled analysis of studies comparing simultaneous ulnar and radial artery compression vs isolated radial artery compression for the endpoint of hemostasis time.

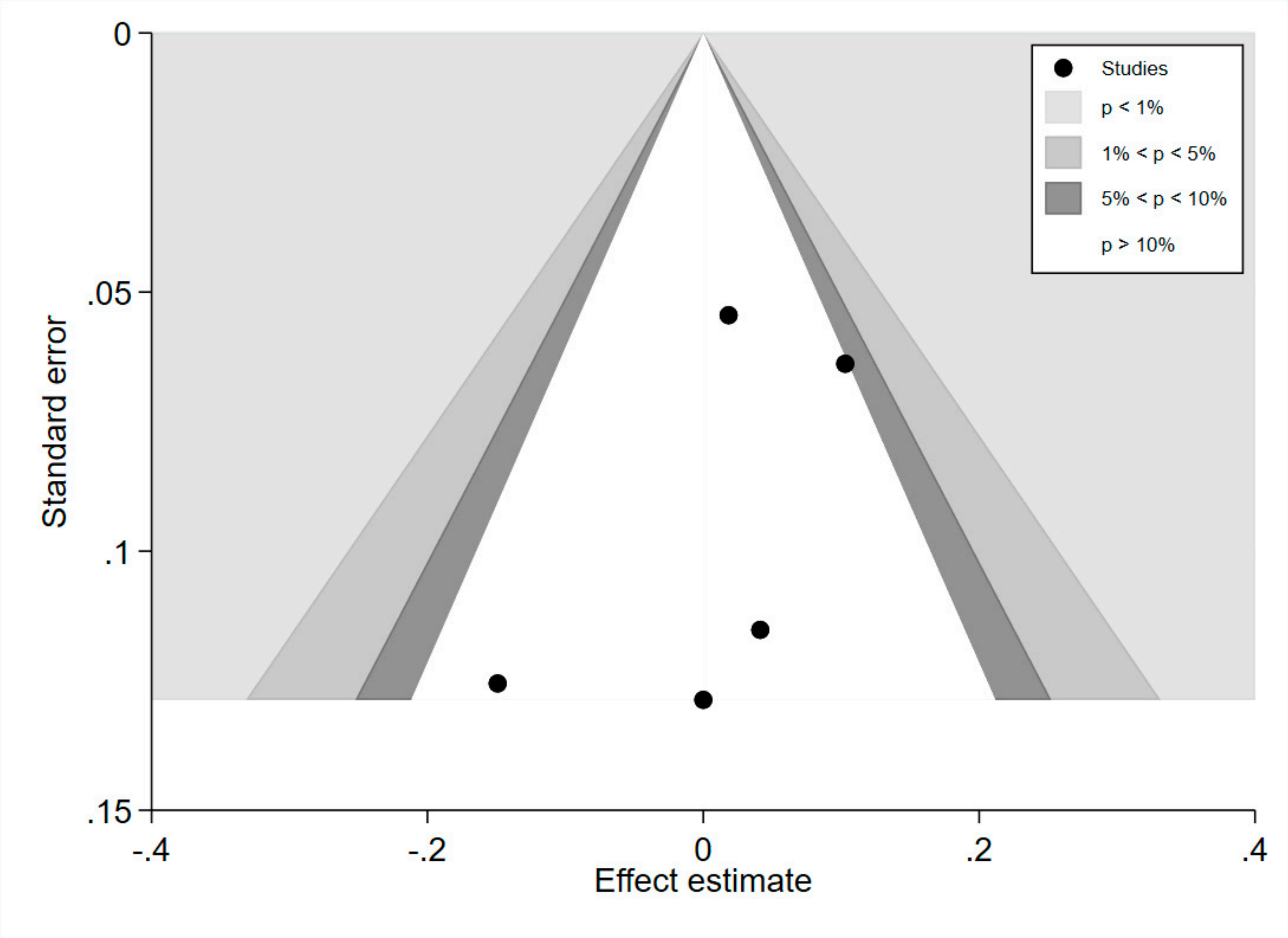

Figure S8: Random-effects meta-regression showing the effect of smoking on the odds ratio of the primary endpoint overall RAO of simultaneous ipsilateral ulnar and radial artery compression versus isolated radial artery compression.

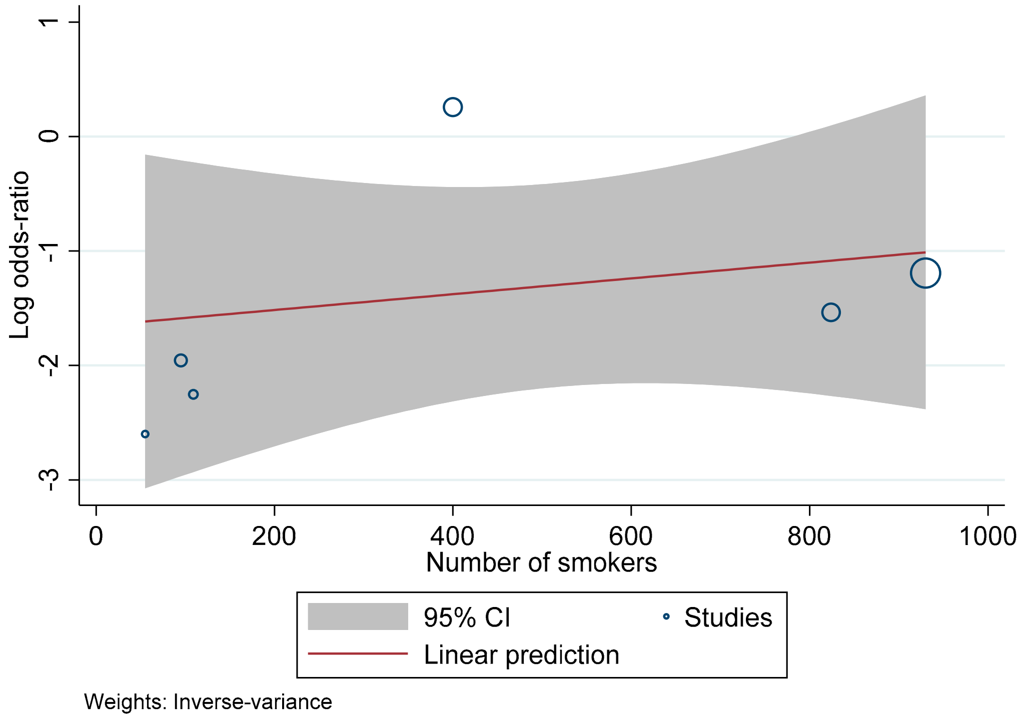

Figure S9: Pooled analysis of studies comparing simultaneous ipsilateral ulnar and radial artery compression versus isolated radial artery compression. Forest plot reporting trial-specific and summary odds ratios with 95% confidence interval (CI) for the endpoint of overall RAO according to study design.

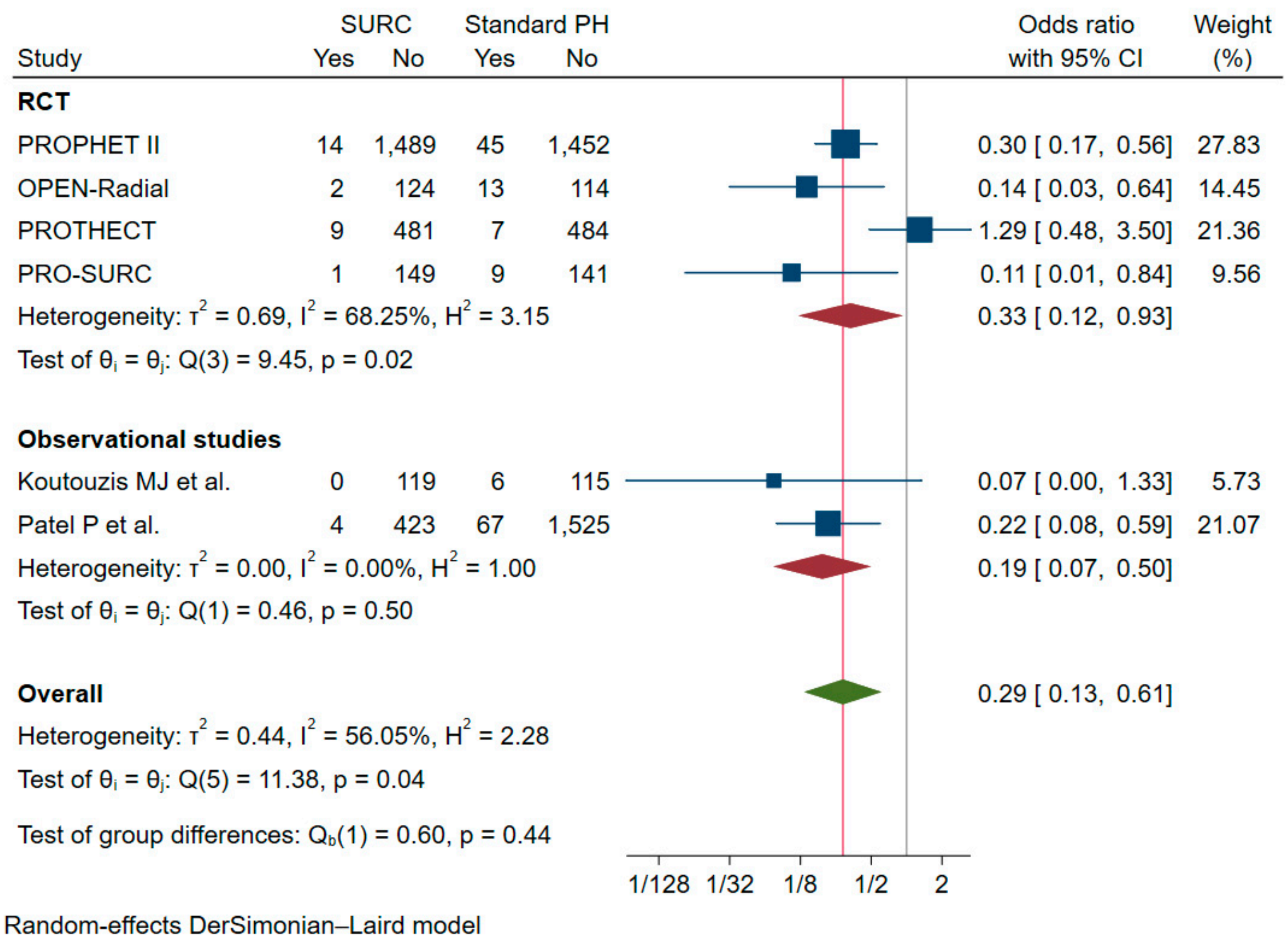

Figure S10: Pooled analysis of studies comparing simultaneous ipsilateral ulnar and radial artery compression versus isolated radial artery compression. Forest plot reporting trial-specific and summary odds ratios with 95% confidence interval (CI) for the endpoint of overall RAO according to the access sheath size

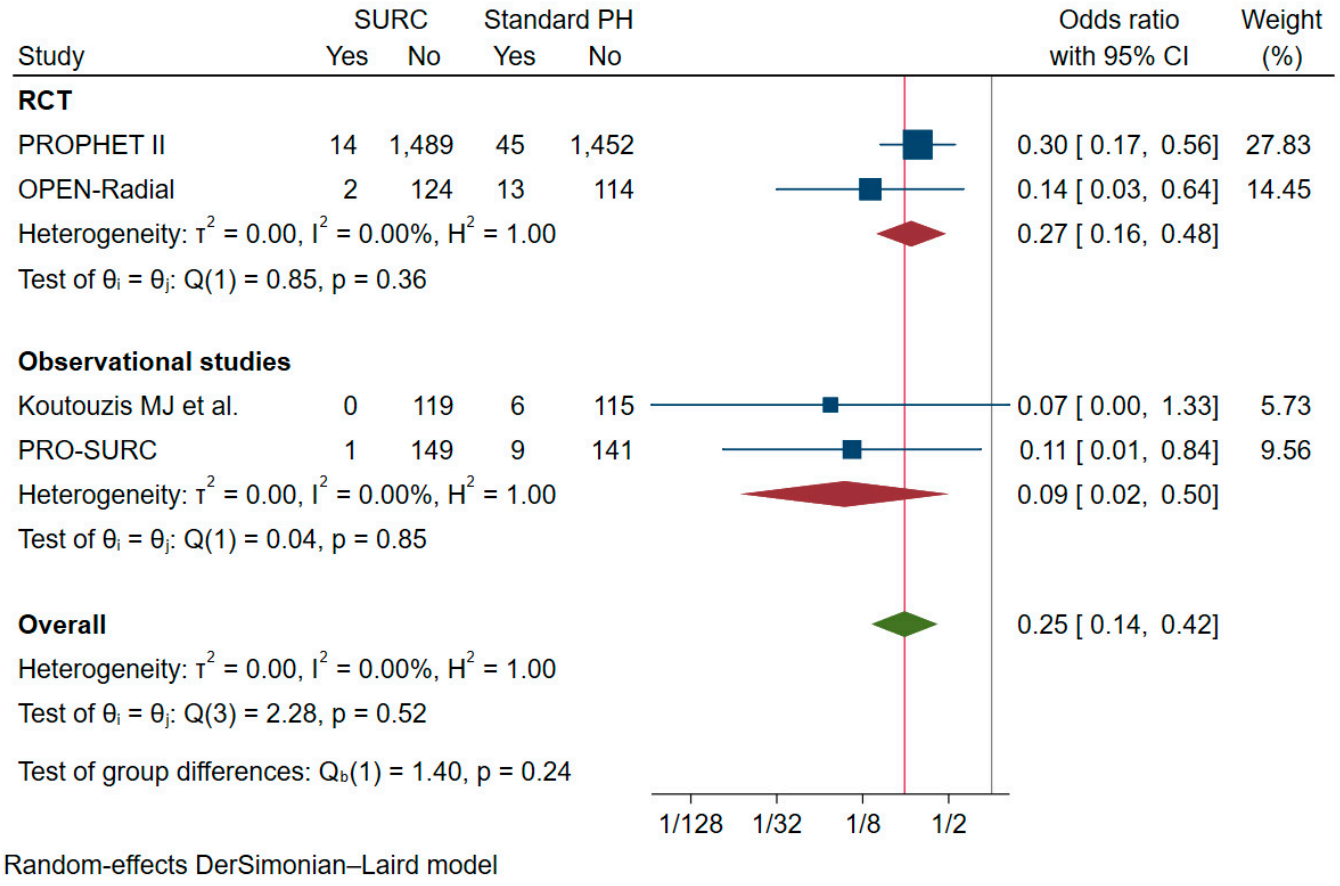

Figure S11: Pooled analysis of studies comparing simultaneous ipsilateral ulnar and radial artery compression versus isolated radial artery compression. Forest plot reporting trial-specific and summary odds ratios with 95% confidence interval (CI) for the endpoint of overall RAO according to the use of dedicated versus non-dedicated ulnar artery compression devices.

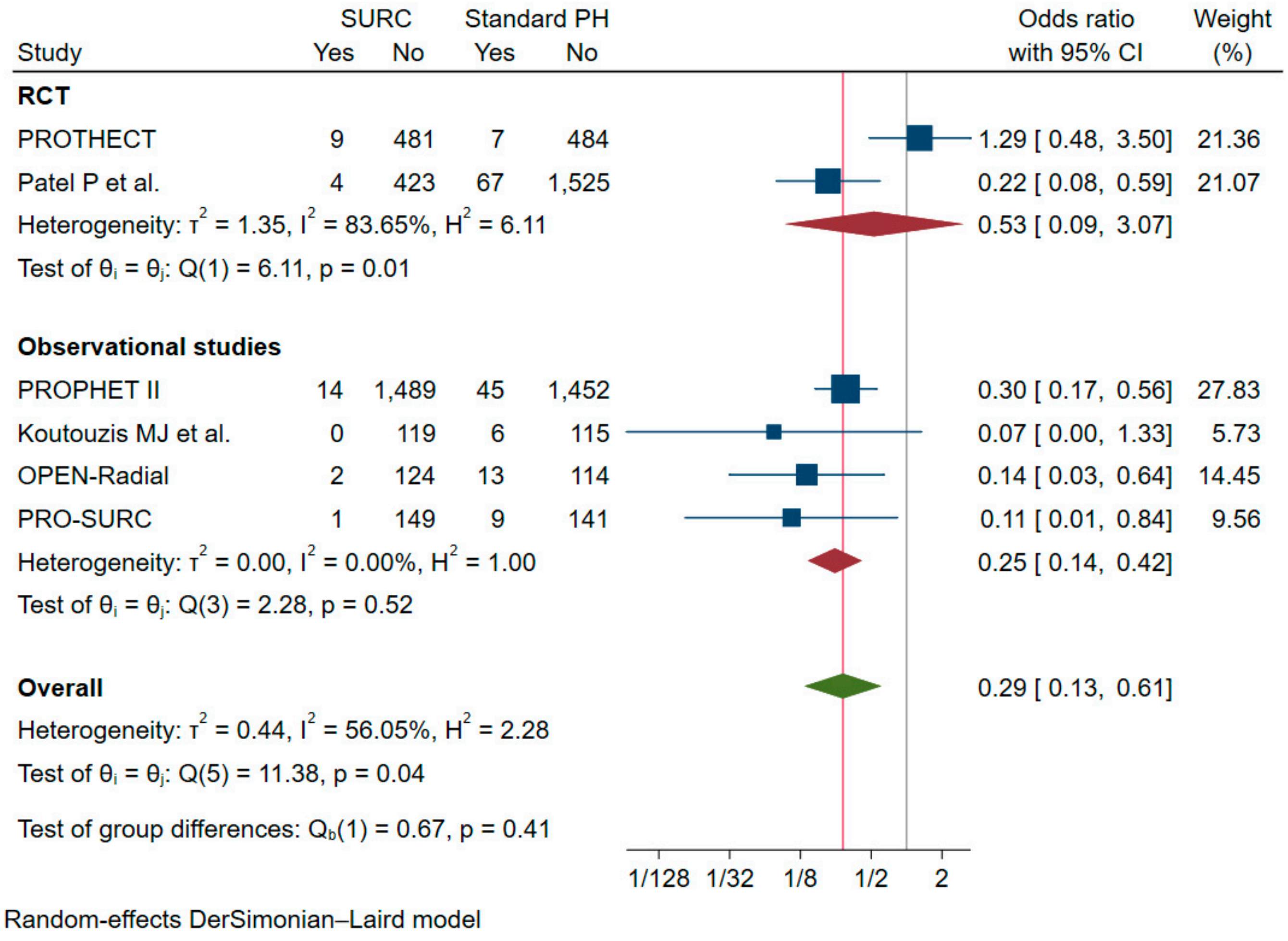

Figure S12: Leave-one out sensitivity analysis for the primary endpoint of overall RAO. Forest plot of summary odds ratios (RR) with 95% confidence interval (CI) after removing one study in turn.

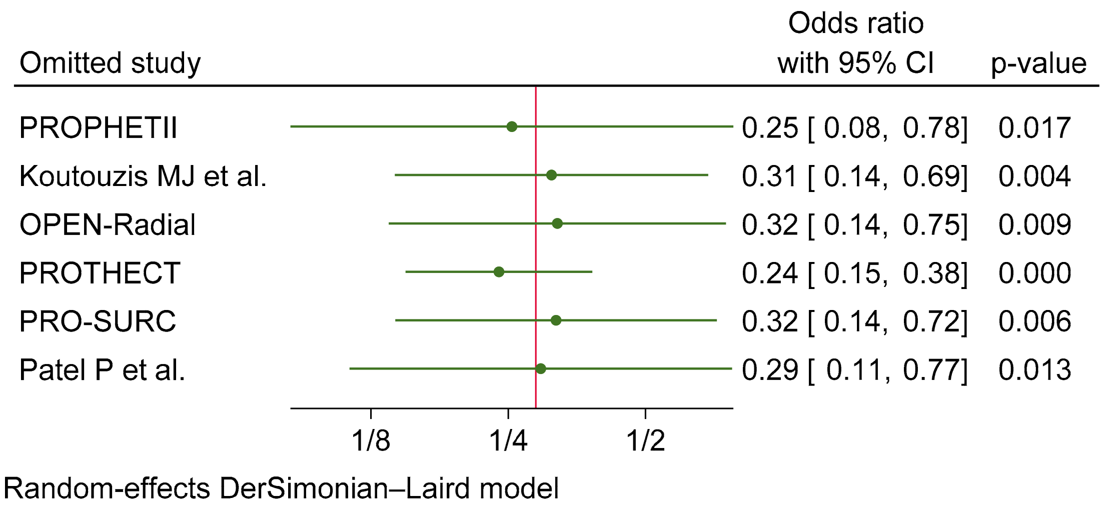

Figure S13: Leave-one out sensitivity analysis for the secondary endpoint of in-hospital RAO. Forest plot of summary odds ratios (OR) with 95% confidence interval (CI) after removing one study in turn.

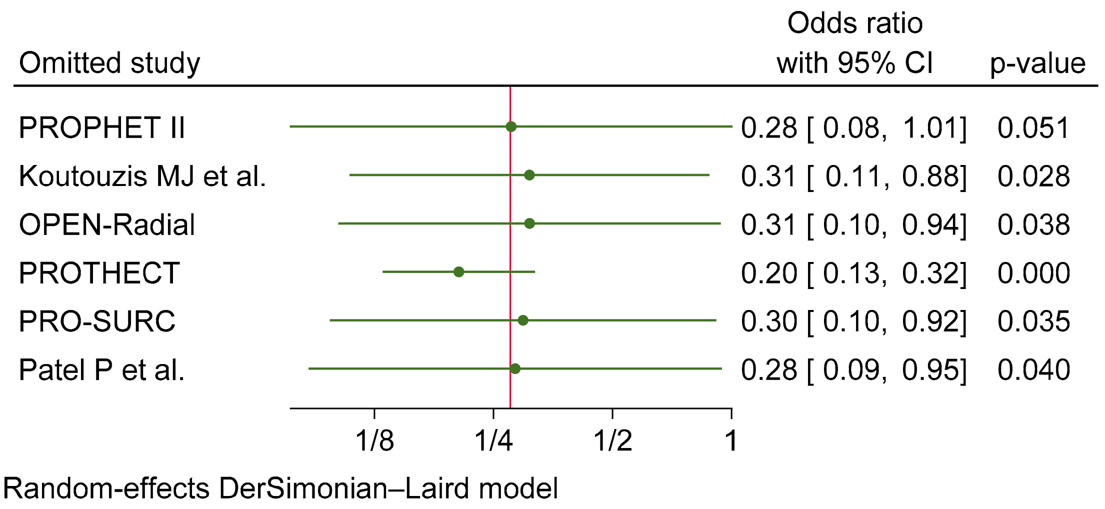

Figure S14: Leave-one out sensitivity analysis for the secondary endpoint of unsuccessful patent hemostasis. Forest plot of summary odds ratios (OR) with 95% confidence interval (CI) after removing one study in turn.

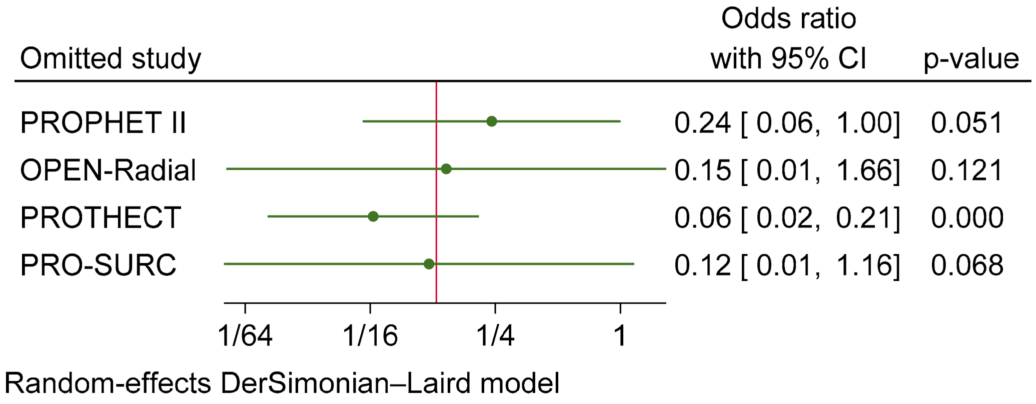

Figure S15: Trial sequential analysis of the primary endpoint using random-effects meta-analysis, based on low-bias risk computed relative risk reduction, a control event incidence with alpha 5% and statistical power of 80%

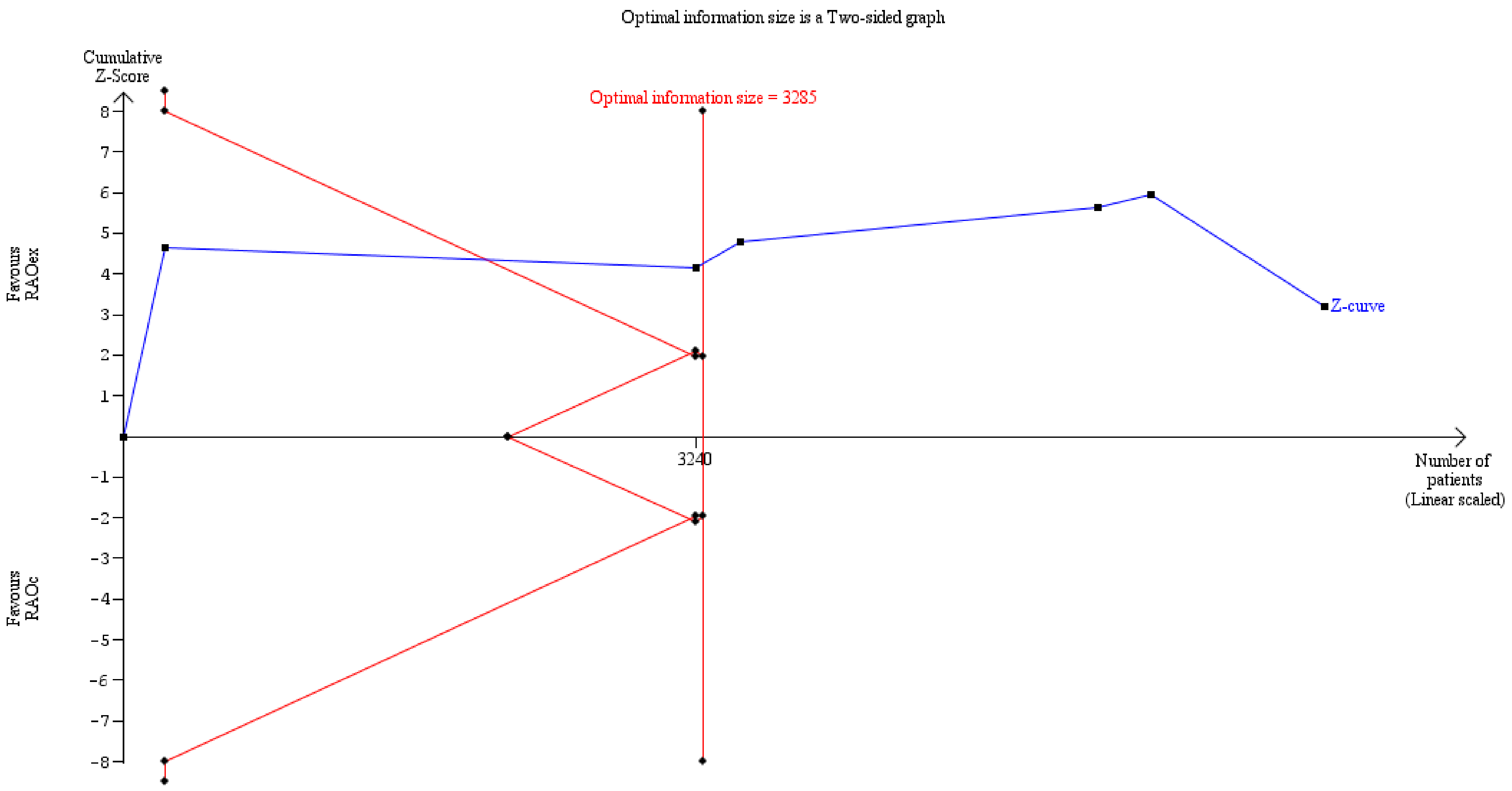

Supplement: Supplementary file 1 [file jcm-11-07013-s001.zip › jcm-2059887-supplementary.pdf]
